# Impact of indoor residual spraying with pirimiphos-methyl (Actellic 300CS) on entomological indicators of transmission and malaria case burden in Migori County, western Kenya

**DOI:** 10.1038/s41598-020-61350-2

**Published:** 2020-03-11

**Authors:** Bernard Abong’o, John E. Gimnig, Stephen J. Torr, Bradley Longman, Diana Omoke, Margaret Muchoki, Feiko ter Kuile, Eric Ochomo, Stephen Munga, Aaron M. Samuels, Kiambo Njagi, James Maas, Robert T. Perry, Christen Fornadel, Martin J. Donnelly, Richard M. Oxborough

**Affiliations:** 1 Abt Associates, PMI VectorLink Project, White House, Milimani, Ojijo Oteko Road, P.O. Box 895-40123, Kisumu, Kenya; 2 Liverpool School of Tropical Medicine, Pembroke Place, Liverpool, L3 5QA UK; 3 Centre for Global Health Research, Kenya Medical Research Institute, P.O. Box 1578, Kisumu, Kenya; 4 Division of Parasitic Diseases and Malaria, Center for Global Health, Centers for Disease Control and Prevention, Atlanta, GA 30333 USA; 5 Kenya National Malaria Control Programme (NMCP), Ministry of Health, PO Box 19982, Kenyatta National Hospital, Nairobi, 00202 Kenya; 6 The United States Presidents Malaria Initiative (PMI), US Embassy Nairobi, United Nations Avenue, Nairobi, Kenya; 7 The United States Presidents Malaria Initiative (PMI), US Agency for International Development, Washington, DC USA; 8 PMI VectorLink Project, Abt Associates 6130 Executive Blv, Rockville, MD 20852 USA

**Keywords:** Population dynamics, Entomology, Epidemiology

## Abstract

Indoor residual spraying (IRS) of insecticides is a major vector control strategy for malaria prevention. We evaluated the impact of a single round of IRS with the organophosphate, pirimiphos-methyl (Actellic 300CS), on entomological and parasitological parameters of malaria in Migori County, western Kenya in 2017, in an area where primary vectors are resistant to pyrethroids but susceptible to the IRS compound. Entomological monitoring was conducted by indoor CDC light trap, pyrethrum spray catches (PSC) and human landing collection (HLC) before and after IRS. The residual effect of the insecticide was assessed monthly by exposing susceptible *An. gambiae* s.s. Kisumu strain to sprayed surfaces in cone assays and measuring mortality at 24 hours. Malaria case burden data were extracted from laboratory records of four health facilities within the sprayed area and two adjacent unsprayed areas. IRS was associated with reductions in *An. funestus* numbers in the intervention areas compared to non-intervention areas by 88% with light traps (risk ratio [RR] 0.12, 95% CI 0.07–0.21, p < 0.001) and 93% with PSC collections (RR = 0.07, 0.03–0.17, p < 0.001). The corresponding reductions in the numbers of *An. arabiensis* collected by PSC were 69% in the intervention compared to the non-intervention areas (RR = 0.31, 0.14–0.68, p = 0.006), but there was no significant difference with light traps (RR = 0.45, 0.21–0.96, p = 0.05). Before IRS, *An. funestus* accounted for over 80% of *Anopheles* mosquitoes collected by light trap and PSC in all sites. After IRS, *An. arabiensis* accounted for 86% of *Anopheles* collected by PSC and 66% by CDC light trap in the sprayed sites while the proportion in non-intervention sites remained unchanged. No sporozoite infections were detected in intervention areas after IRS and biting rates by *An. funestus* were reduced to near zero. *Anopheles funestus* and *An. arabiensis* were fully susceptible to pirimiphos-methyl and resistant to pyrethroids. The residual effect of Actellic 300CS lasted ten months on mud and concrete walls. Malaria case counts among febrile patients within IRS areas was lower post- compared to pre-IRS by 44%, 65% and 47% in Rongo, Uriri and Nyatike health facilities respectively. A single application of IRS with Actellic 300CS in Migori County provided ten months protection and resulted in the near elimination of the primary malaria vector *An. funestus* and a corresponding reduction of malaria case count among out-patients. The impact was less on *An. arabiensis*, most likely due to their exophilic nature.

## Introduction

Over the last two decades, malaria control has been scaled up throughout sub-Saharan Africa with an emphasis on the distribution of long-lasting insecticidal nets (LLINs), targeted application of indoor residual spraying (IRS), and improved diagnostics and case management. As a result, the burden of malaria has declined substantially with a 40% reduction in incidence and a 50% reduction in prevalence between 2000 and 2015. While LLINs contributed an estimated 68% of the decline in malaria prevalence, IRS was responsible for 13%^1^.

The efficacy of insecticide-treated nets was demonstrated in a series of cluster randomized, controlled trials^2^. Formal randomized controlled trials of IRS have also demonstrated the efficacy of IRS^3,4^. Furthermore, there is a long history of programmatic implementation of IRS in many settings of the world which resulted in reduced malaria burden and even elimination in some settings^2^. The use of both LLINs and IRS for malaria control has a direct impact on mosquito bionomics. LLINs and IRS have multiple effects on mosquito populations which may result in reduced malaria transmission including: reduced indoor *Anopheles* densities^5–7^, shifts in vector species composition^8^, changes in the time and location of mosquito biting^9–11^, and changes in host selection^12^, and increases in early exophily^13^.

In western Kenya, vector control included universal coverage of LLINs through periodic mass campaigns and routine distribution to high-risk groups as well as IRS in specifically targeted areas. The first mass LLIN distribution occurred in 2006 and targeted children <5 years of age. Additional distributions aiming for universal coverage occurred in 2011 and 2014 leading to 54% of households in the lake endemic zone having one LLIN for every two residents^14^. The region also bears the highest malaria burden nationally^14–16^. Implementation challenges facing LLINs include incomplete coverage^14,17–19^, widespread pyrethroid resistance^20–23^ and possible changes in vector behaviour^9^.

IRS in western Kenya was based exclusively on pyrethroids until 2012^24–26^. However, spraying was interrupted between 2013 and 2017 due to widespread pyrethroid resistance in local malaria vector populations and the lack of a registered, non-pyrethroid insecticide in the country. In response to widespread pyrethroid resistance, the Kenyan National Malaria Control Programme (NMCP) developed an insecticide resistance management strategy involving the rotation of different non-pyrethroid classes of insecticides used in IRS every two years in endemic and epidemic-prone areas where 80% or more households own one or more LLIN^27^. This is in accordance with global insecticide resistance management strategy aimed at delaying the rise and spread of insecticide resistance to new classes of insecticide while preserving pyrethroids for use in bednets^28^. In 2017, IRS was re-introduced using a microencapsulated formulation of pirimiphos-methyl (Actellic 300CS (capsule suspension)). The insecticide has been reported to be effective against pyrethroid-resistant *Anopheles* mosquitoes^29–31^ and has a relatively long residual effect on sprayed wall surfaces, of up to twelve months^29,30,32^.

Between 2008 and 2012, IRS campaigns in western Kenya were conducted using pyrethroid based insecticides. These campaigns resulted in significantly lower parasitaemia and cases of clinical malaria in IRS districts compared to non-IRS districts^26^. The protective efficacy of ITN plus IRS compared with ITN only in the same area was measured at 62%, whereas, 60% fewer anophelines were observed in the sprayed districts compared to unsprayed districts post-IRS^25^. *Anopheles funestus* accounted for 1.7% of total anophelines collected in the sprayed areas before IRS and 2.7% after IRS^25^, an indication that the species was not affected by spraying. Following reports of increasing insecticide resistance, the malaria vector control policy changed to the use of non-pyrethroid insecticides for IRS. IRS was reintroduced in 2017 with an organophosphate, Actellic 300CS. We sought to determine efficacy of the Actellic CS based IRS campaign on entomological and epidemiological indices against a background of moderate to high coverage of pyrethroid LLINs^14^ and extensive pyrethroid resistance.

## Methods

### Study sites

**Figure 1:**
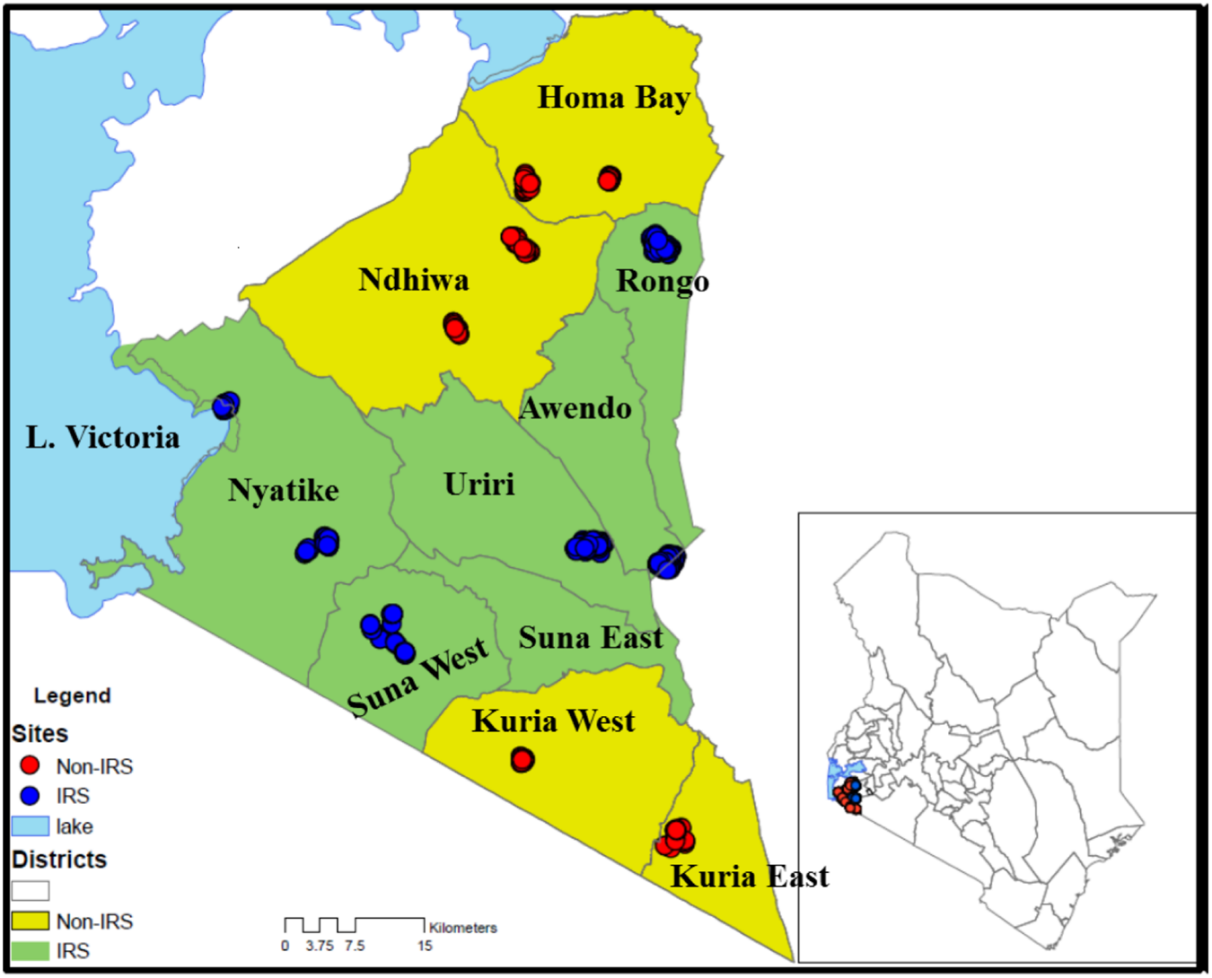
Map of Kenya, showing study sites in western Kenya with the names of sub-counties. Yellow shading represents non-intervention sites and red dots represent sampled houses. The green shading is the intervention site with the blue dots representing sampled houses (The map was created on ArcGIS 10.2.2).

Entomological monitoring was conducted in 12 villages in Migori (−1.0667S; 34.4667E) and Homa Bay (−0.5396S; 34.4565E) counties from July 2016 to February 2018. Six IRS intervention sub-counties were in Migori County, and six control sub-counties were in neighbouring Homa Bay County (n = 4) and unsprayed areas of Migori County (n = 2) (Fig. 1). The residents in the study area are mainly of the Luo ethnic group and are subsistence farmers with a few growing cash crops such as sugar cane and tobacco. Residents mostly live in small houses, clustered into family social units called compounds. The region has bimodal peaks of rainfall with the long rains between April and June and short rains in October and November. The Lake Victoria region of western Kenya is malaria endemic; the most recent Malaria Indicator Survey in 2015 documented a malaria prevalence of 27% by microscopy. The last mass net campaign in the region was conducted in April 2017 with DawaPlus 2.0, a deltamethrin long-lasting (coated) insecticidal net distributed in this area. Though 87% of households own at least one LLIN and 60% own more than one LLIN, only 54% of households have an adequate number of nets, defined as one LLIN for every two residents. Among residents of western Kenya, 68.4% reported to have slept under any net the night before the survey while 66.9% had slept under LLINs^14^. *Anopheles funestus*, *An. arabiensis* and *An. gambiae* s.s. are the main malaria vectors in the region^33,34^. Insecticide resistance among major malaria vectors in Kenya has been reported for pyrethroids, carbamates, and organochlorines^35^. The reported resistance mechanisms include both target site mutations and increased activity of enzymes involved in metabolic detoxification^21–23^.

#### IRS campaign

IRS was conducted in February-March 2017. A total of 212,029 houses in Migori County were sprayed, representing coverage of 97.7% of houses sprayed against houses found. The campaign covered a population of 906,388 people, including 16,932 pregnant women and 127,157 children below five years of age^36^.

#### Vector surveillance

Vector surveillance was conducted in the twelve villages from July 2016 to February 2018. Houses were randomly selected in each village every month for mosquito collections by PSC and indoor CDC light trap (CDC-LT). Household information including roof type, wall type, open or closed eaves, the presence of nets, number of people that slept under a net the previous night and those that did not, and the presence of cattle were collected on a tablet computer. The mosquito density for each method were expressed as the mean number of mosquitoes caught per house per collection visit.

Indoor-resting mosquitoes were collected between 07:00 and 11:00 by PSC in five houses per site per month. PSCs were done by laying white sheets on the floor and over the furniture within the house. Two collectors, one inside the house and another outside, sprayed around the eaves with 0.025% pyrethrum emulsifiable concentrate mixed with 0.1% piperonyl butoxide (supplied by the Kenya Pyrethrum Board) in kerosene. The collector inside the house then sprayed the roof space. The house was closed for 10–15 minutes after which knocked-down mosquitoes were collected from the sheets and transferred to the laboratory in scintillation vials containing 70% ethanol.

Indoor host-seeking mosquitoes were collected by CDC-LT in 10 houses per site once per month. A single 12-volt CDC-LT was hung in each house in the sleeping area, approximately 1.5 meters from the floor, adjacent to an occupied bed net owned by a member of the household. The traps were run from 18:00 to 07:00 the following morning. The trapped mosquitoes were transferred into paper cups and transported to the laboratory for further analysis.

Human landing catches (HLC) were used to assess biting time and location (indoor vs outdoor) of the local vector population before and after spraying. HLC was done during the short rains pre-IRS in November 2016, and after the long rains in June 2017. Collections were performed at six sites used for routine surveillance, two in non-IRS areas and four IRS areas. In each site, five houses were randomly selected, and collections were performed for five consecutive nights in each house once before and after IRS.

During HLC, one volunteer sat outside within 5 meters from the house, and another sat inside the house in the living room. Collectors kept their trousers folded to knee length and aspirated any mosquitoes landing on their lower legs. Each house had a team of six collectors, each working in pairs during one of three six-hour shifts running from 17:00 to 11:00 the next morning. Collections were performed for 45 minutes, and the collectors rested for 15 minutes in each collection hour. The collectors recorded the location of members of the household observed at the end of each hour as either outdoor, in the living room, or in the bedroom. Collected mosquitoes were separated by time and location of collection and sustained on 10% sugar solution before being transported to the laboratory for analysis. Estimation of exposure of individuals to bites by *An. funestus* was performed using models previously described by Seyoum *et al*.^37^.

#### Persistence of insecticidal activity on sprayed walls

To assess the persistence of insecticidal activity on sprayed walls following IRS, WHO cone bioassays^38^ were conducted each month using laboratory-reared, 2–5 day old, non-blood fed susceptible colony of *An. gambiae* s.s. Kisumu strain. Mosquitoes were exposed in 10 randomly selected sprayed houses, seven with mud walls and three with cement walls, in each of four sub-counties in Migori county. Exposures were performed with ten mosquitoes per cone each month in the same houses at three different heights (0.5 m, 1 m, and 1.5 m) from the floor for 30 minutes, on three different walls of the living room of each sprayed house. A control cone with ten mosquitoes was set on an unsprayed plywood board outside of each sprayed house in a shaded area close to the house. Temperature and relative humidity were recorded at every house where mosquitoes were exposed.

#### Insecticide resistance monitoring

WHO insecticide susceptibility tests were performed in Rongo, Nyatike, Awendo and Uriri sub-counties in Migori County (IRS sites) and Homa Bay and Ndhiwa sub-counties in Homa Bay County (no IRS). Larval stages of *An. gambiae* s.l. were collected from Homa Bay, Ndhiwa, Rongo, Nyatike and Awendo sub-counties before IRS and in Homa Bay, Ndhiwa and Rongo after IRS. Larval samples were collected mainly from borrow pits, tire tracks, farmlands and trenches. The collected larvae were raised to three-day-old adults before testing. Adult *An. funestus* were also collected by hand aspiration inside houses for insecticide resistance tests, as larvae were difficult to find. Collections were performed in Homa Bay and Ndhiwa, Rongo, Awendo and Uriri sub-counties before IRS. However, after IRS few adult mosquitoes were in the sprayed sites, so no *An. funestus* s.l. were available for testing from these areas. CDC bottle intensity assay was used to assess intensity of resistance for *An. arabiensis* from Awendo, Ndhiwa and Homa Bay counties. Three-day-old adults raised from field-collected larvae were exposed in bottle coated with doses of 1X, 2X, 5X and 10X of permethrin. Survival frequency of *An. arabiensis* to the different doses was determined at 30 minutes exposure period.

Insecticide resistance status was assessed using the WHO diagnostic concentrations of deltamethrin (0.05%), permethrin (0.75%), pirimiphos-methyl (0.25%) and alpha-cypermethrin (0.05%). All papers were prepared by the WHO collaborating centre, Universiti Sains Malaysia. The WHO bioassay was done using 2- to 5-day-old *An. gambiae* s.l. emerging from collected larvae or by direct exposure of field-collected adult *An. funestus* since these were difficult to collect as larvae. At least 100 mosquitoes (four replicates of 25) of each species were exposed to each insecticide per sub-county. The samples were then transferred to a holding tube, provided with cotton wool soaked in 10% sugar solution and held for 24 hours. Mortality was scored 24 hours after exposure.

#### Mosquito species identification, sporozoite infection and blood meal identification

All *Anopheles* collected were identified morphologically to species using the keys of Gillies and DeMeillon or Gillies and Coetzee^39,40^. The physiological status was determined by observation of the abdomen to classify female mosquitoes as either blood-fed, gravid, half gravid or unfed. Female mosquitoes were dissected into three parts for various procedures: heads and thoraces were used for determination of *Plasmodium falciparum* sporozoite infection by enzyme-linked immunosorbent assay (ELISA) using the MR4 Methods in *Anopheles* Research adapted from Wirtz *et al*.^41,42^; the abdomens of blood-fed females were used to determine the source of mosquito blood meals by targeting *cytochrome b* protein using a multiplexed PCR protocol^43^, with slight modifications. The legs and wings were used in PCR analyses to identify to species level members of the *An. gambiae* species complex and *Anopheles funestus* group^44^. All mosquitoes morphologically identified as *An. gambiae* s.l. and 20% of randomly selected *An. funestus* s.l., were analyzed by PCR each month. This approach was done due to the greater number of *An. funestus* collected and based on previous studies in the area showing that *An. gambiae* and *An. arabiensis* are found in sympatry, while *An. funestus* s.s. was the only member of the species group routinely collected^33,34^.

#### Health facility surveillance

Health facility laboratory data were collected from Rongo, Uriri, and Macalder sub-county hospitals within Migori County (IRS) and Marindi health centre and Ndhiwa sub-County hospital in Homa Bay County (No IRS). The facilities were chosen based on proximity to entomological surveillance sites, availability of health records and catchment area as falling within either IRS or non-IRS area. Febrile cases were tested by health facility staff using light microscopy as part of routine health care and data were recorded in registers provided by the Kenya Ministry of Health. Data were abstracted from laboratory registers of the selected health facilities for the period from January 2015 until June 2018. Each page of the register was photographed using a smartphone camera, and the photographs converted to PDF files using CamScanner-Phone PDF creator, (INTSIG Information Co., Ltd). To ensure confidentiality, the column containing the patient’s name was covered when taking the photograph. The PDF copies were then printed and filed.

#### Data management and analysis

Field entomological data collection used Open Data Kit software (ODK) run on tablets with an interface designed to limit data entry errors. Data entry screens used drop-down menus and automatic data checks to reduce errors. Each house sampled received a unique code and a study number. Individual mosquitoes were placed in Eppendorf tubes labeled with pre-printed barcodes and linked to the field data by house code and a unique study number. Results of additional testing, including sporozoite ELISA, species identification by PCR and blood meal analysis, were linked to an individual mosquito by the unique barcode label. Individual patient records included date of testing, age, gender, village, clinical diagnosis, test performed, and test results from scanned copies of health facility registers. Data were entered into a Microsoft Access database.

Data analysis was performed using R statistical software version 3.4.1 or SAS version 9.4. The risk ratio (RR) was used to assess the statistical significance of differences in mosquito densities pre and post IRS, between intervention and non-intervention sites. Data were fitted using Generalized Linear Mixed Effects Statistical Models (GLMMs). Since the data were over-dispersed, we used the package Generalized Linear Mixed Models using Template Model Builder (glmmTMB) or PROC GLIMMIX, to fit negative binomial distribution models for the analysis of mosquito numbers. The numbers of female *Anopheles* mosquitoes were assessed as a function of the period of collection (before or after IRS) and intervention status (sprayed or non-sprayed) as a fixed effect, while village was treated as a random effect. To obtain the risk ratios (RR) and confidence intervals, we exponentiated the model coefficients. Models were adjusted for reported net use, the presence of open eaves, and the presence of cattle on the compound. A test of interaction was performed to compare differences in estimates of mosquito numbers between the period of mosquito collection and intervention status^45^. Conditional estimates of the change in mosquito densities pre- and post-IRS conditional on the IRS or non-IRS County were generated. To analyse *Anopheles* species proportions pre-and post-IRS in intervention and non-intervention areas for each trapping method, a binomial GLM model was used. The model was also used to analyse sporozoite rates (proportion of sporozoite ELISA tests that are positive) and human biting rates between intervention and non-intervention sites, before and after IRS and proportions of the types of mosquito host blood meals.

To detect changes in numbers of malaria cases before and after IRS within each health facility Auto-Regressive Integrated Moving Average (ARIMA) analysis was performed. Data from each facility was analysed using the “Time Series Analysis” (TSA)^46^ and “Alternative Time Series Analyses” (aTSA)^47,48^ packages in R. The ARIMA model was derived by observation of the autocorrelation and partial-autocorrelation functions to determine the most parsimonious solution of the “order” (p), “differencing” (d), and “moving-average” (q) parameter values. The model was then regressed on the absence (prior to) or presence of IRS in the village to estimate the value of the number of positive malaria cases prior to, and during the period of IRS.

#### Ethical considerations

The study was approved by the Kenya Medical Research Institute/ Scientific and Ethics Review Unit (KEMRI/SERU), number 2776 and by CDC through a reliance agreement with KEMRI/SERU (CDC IRB 6728). Individuals participating in HLC gave informed consent. They were screened for malaria before the start of the study and treated if positive. Collectors were placed on mefloquine malaria prophylaxis, (Mephaquin, Acino Pharma AG, Switzerland) one week before collections began, with repeat doses once every week through the collection period, until four weeks after collections ended. During routine mosquito collections, verbal consent was sought from the household head to use CDC-LT and PSC in their compound. All methods were performed in accordance with relevant guidelines and regulations.

## Results

### Vector species composition and seasonality

The mean number of *An. funestus* and *An. gambiae*. s.l. found in indoor CDC-LT and PSCs are presented by IRS status and period (pre- or post-IRS) in Table 1. The number of each species of mosquito collected by the two different methods was compared using negative binomial regression models incorporating IRS status, period, an interaction between IRS status and period, net use, the presence of open eaves and the presence of cattle on the compound (Supplemental Tables 1–4). For all models except for the *An. gambiae* s.l. collected by PSC, the interaction term was statistically significant indicating a differential effect of period based upon the IRS status. Conditional estimates of the effect of period controlling for IRS status are provided in Table 1.

**Table 1:** Comparison of mean numbers of *An. funestus* and *An. arabiensis* collected indoors by CDC-LTs and PSCs pre- and post-IRS in intervention and non-intervention areas. Risk ratios of post- versus pre-IRS periods conditional on intervention status are also provided for each species and collection method. Models include terms for IRS status, pre/post spray period and an interaction term (Table 1). See Supplemental Tables 1–4 for full models.

*Anopheles* Species	Collection Method	IRS Status	Level	Mean	Risk Ratio	Lower CL	Upper CL	P-value
*Anopheles funestus*	Light trap	IRS	Post-Spray	0.05	0.12	0.07	0.19	**<0.001**
			Pre-Spray	0.45	Ref			
		Non-IRS	Post-Spray	0.88	0.98	0.69	1.38	0.899
			Pre-Spray	0.92	Ref			
	PSC	IRS	Post-Spray	0.04	0.04	0.02	0.07	**<0.001**
			Pre-Spray	0.99	Ref			
		Non-IRS	Post-Spray	1.05	0.64	0.41	1.00	0.052
			Pre-Spray	2.05	Ref			
*Anopheles arabiensis*	Light trap	IRS	Post-Spray	0.19	1.39	0.78	2.47	0.266
			Pre-Spray	0.10	Ref			
		Non-IRS	Post-Spray	0.21	3.06	1.59	5.92	**0.001**
			Pre-Spray	0.05	Ref			
	PSC	IRS	Post-Spray	0.24	0.60	0.33	1.09	0.093
			Pre-Spray	0.52	Ref			
		Non-IRS	Post-Spray	0.41	1.64	0.87	3.09	0.123
			Pre-Spray	0.27	Ref			

The number of *An. funestus* collected in light traps in intervention sites were significantly lower in the post-IRS compared to pre-IRS period (RR = 0.12, 95% CI: 0.07–0.19, P < 0.001). No significant difference in the mean number of *An. funestus* was observed in the non-intervention sites between pre- and post-IRS (IRR = 0.98, 95% CI: 0.69–1.38, p = 0.899). A statistically significant difference-of-differences between the period of mosquito collection and intervention status was observed based on the statistically significant interaction term (RR = 0.12, 95% CI: 0.07–0.21) (Supplemental Table 1). From PSC collections, significantly fewer numbers of *An. funestus* were observed in both IRS and non-IRS sites in the post-IRS period compared to pre-IRS period (RR = 0.04, 95% CI: 0.02–0.07, p < 0.001). The number of *An. funestus* in the non-IRS area also declined but the conditional difference between pre-IRS and post-IRS was not statistically significant (RR = 0.64, 95% CI: 0.41–1.00, p = 0.052). A statistically significant difference-of-differences was observed between period of mosquito collection and intervention status post-IRS indicating a stronger decline in the IRS sites compared to the non-IRS sites (RR = 0.06, 95% CI: 0.03–0.13, p < 0.001) (Supplemental Table 2).

The mean numbers of *An. arabiensis* collected in indoor CDC-LTs in both intervention and non-intervention sites increased in the post-IRS compared to pre-IRS period with a statistically significant increase in the non-IRS sites (IRS sites: RR = 1.39, 95% CI: 0.78–2.47, p = 0.266; non-IRS sites: RR = 3.06, 95% CI: 1.59–5.92, p = 0.001). The conditional estimates are provided in Table 1 although the interaction term was not significant (RR = 0.45, 95% CI: 0.2–1.01, p = 0.052) (Table 1) indicating the increase was not statistically greater in the non-IRS sites than the IRS sites (Supplemental Table 3).

The mean numbers of *An. arabiensis* collected by PSC in the intervention sites were not significantly different in the post-IRS compared to pre-IRS period (RR = 0.60, 95% CI: 0.33–1.09, p = 0.093). For the non-IRS areas, the number of *An. arabiensis* collected by PSC increased although not significantly (RR = 1.64, 95% CI: 0.87–3.09, p = 0.123). Although no significant difference in the mean numbers of *An. arabiensis* was observed pre- and post-IRS in either the IRS or the non-IRS sites, a statistically significant difference-of-differences was observed between the period of mosquito collection and intervention status indicating a significant difference between the IRS and non-IRS areas after IRS implementation (RR = 0.36, 95% CI: 0.16–0.82, p = 0.015) (Supplemental Table 4).

**Figure 2:**
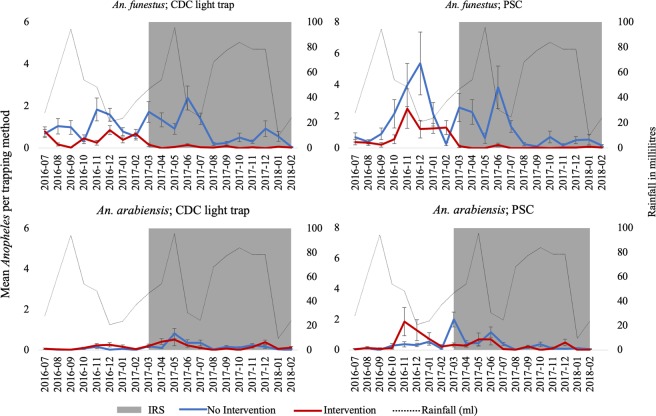
Mean number of observed female *Anopheles* (means ± std error) per trap-night per month in indoor CDC light trap and PSC before and after IRS in sprayed and unsprayed areas. The grey shaded area is the period post-spray with residual efficacy above 80%. The primary scale shows *Anopheles* density while the secondary scale shows rainfall in milliliters. Sixty trap-nights for CDC-LT and 30 for PSC per study arm per month. Standard errors were calculated by dividing the standard deviation by the square root of the sample size, a function was developed in R that generated the standard error around each mean.

The mean number of *An. funestus* collected by CDC-LT and PSC in both intervention and non-intervention areas varied by month, with the highest numbers collected during the short rainy season before IRS (Nov–Dec 2016) and during the long rainy season in the unsprayed area (March–June 2017) (Fig. 2). After IRS, the mean numbers collected by both CDC-LT and PSC in the intervention areas remained low, with no seasonal variation throughout the study period. The mean number of *An. arabiensis* collected by either method was lower compared to *An. funestus* with little monthly variation before and after IRS. No clear difference was observed in the seasonality of *An. arabiensis* before and after IRS (Fig. 2).

**Figure 3:**
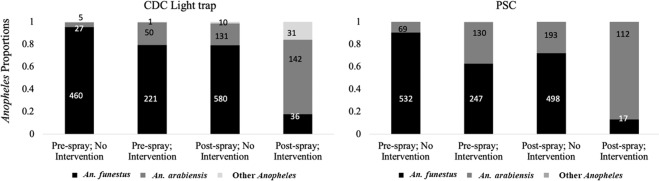
Proportions of *Anopheles* species collected by CDC-LT and PSC before and after IRS in sprayed and unsprayed regions.

Differences in *Anopheles* species composition were observed in intervention areas before and after IRS. In both CDC-LT and PSC collections, *An. funestus* comprised over 80% of the total *Anopheles* collected in both intervention and non-intervention sites before IRS. While *An. funestus* remained predominant in non-intervention sites after IRS, *An. arabiensis* formed the bulk of all *Anopheles* collected in the intervention sites after IRS (Fig. 3).

#### Insecticide decay rate and insecticide resistance monitoring

Mortality rates of susceptible *An. gambiae* s.s females were over 80% up to 10 months post-IRS across all study sites on both mud and concrete walls as measured by WHO cone assay (Supplemental Fig. 1). Using WHO tube bioassays both *An. funestus* (Supplemental Fig. 2A) and *An. arabiensis* (Supplemental Fig. 2B) before IRS and *An. arabiensis* after IRS (Supplemental Fig. 2C) were fully susceptible to pirimiphos-methyl and bendiocarb but resistant to the pyrethroids, deltamethrin, permethrin, and alpha-cypermethrin. Survival frequencies of *An. arabiensis* to different doses of permethrin varied between sites (Supplemental Fig. 3).

#### Factors affecting *Anopheles* numbers

Table 2 presents data showing modelled estimates of the effect of net use, open eaves, and presence of cattle in the compound on the indoor occurrence of *An. funestus* and *An. arabiensis* in sprayed and unsprayed houses, measured by CDC-LT and PSC collections. For *An. funestus*, significantly fewer were collected by light traps in houses with completely closed eaves (RR = 0.68, 95% CI: 0.48–0.96, p = 0.030) while significantly more were collected from houses where cattle were kept on the compound (RR = 1.62, 95%CI: 1.22–2.13, p = 0.001). No other comparisons were statistically significant. By PSC, there were again significantly more *An. funestus* collected in households where cattle were kept on the compound (RR = 1.63, 95% CI: 1.12–2.35, p = 0.010). There were significantly more *An. funestus* in houses where some but not all residents used a net the previous night compared to houses where no one used a net (RR = 2.02, 95% CI: 1.13–3.59, p = 0.017). No other comparisons were statistically significant.

**Table 2:** Model estimates comparing the mean number of indoor *An. funestus* and *An. arabiensis* collected, by collection type, eave type, net use, and presence of cattle in intervention and non-intervention areas. Models include terms for IRS status, pre/post spray period and an interaction term (Table 1). See Supplemental Tables 1–4 for full models.

Species	Collection Method	Parameter	Level	Proportion of Houses	Risk Ratio	Lower CL	Upper CL	t-value	P-value
*Anopheles funestus*	Light Trap	Net Use	All under net	0.47	1.11	0.77	1.6	0.576	0.565
			Some under net	0.09	1.2	0.77	1.86	0.816	0.415
			None under net	0.44	Ref				
		Eaves	Closed	0.20	0.68	0.48	0.96	−2.174	**0.030**
			Partially open	0.10	0.84	0.56	1.27	−0.817	0.414
			Open	0.70	Ref				
		Cattle	Yes	0.71	1.62	1.22	2.13	3.395	**0.001**
			No	0.29	Ref				
	PSC	Net Use	All under net	0.43	0.96	0.61	1.5	−0.187	0.852
			Some under net	0.08	2.02	1.13	3.59	2.383	**0.017**
			None under net	0.49	Ref				
		Eaves	Closed	0.18	0.8	0.5	1.3	−0.889	0.374
			Partially open	0.12	1.08	0.64	1.83	0.291	0.771
			Open	0.70	Ref				
		Cattle	Yes	0.71	1.63	1.12	2.35	2.583	**0.010**
			No	0.29	Ref				
*Anopheles arabiensis*	Light Trap	Net Use	All under net	0.47	1.95	0.99	3.84	1.94	0.052
			Some under net	0.09	2.17	1.02	4.62	2.008	**0.045**
			None under net	0.44	Ref				
		Eaves	Closed	0.20	0.57	0.33	0.96	−2.131	**0.033**
			Partially open	0.09	0.78	0.43	1.42	−0.814	0.416
			Open	0.70	Ref				
		Cattle	Yes	0.71	1.33	0.89	1.98	1.383	0.167
			No	0.29	Ref				
	PSC	Net Use	All under net	0.43	1.61	0.9	2.87	1.594	0.111
			Some under net	0.08	1.85	0.89	3.84	1.652	0.099
			None under net	0.49	Ref				
		Eaves	Closed	0.18	0.34	0.18	0.67	−3.165	**0.002**
			Partially open	0.11	0.60	0.32	1.13	−1.583	0.114
			Open	0.70	Ref				
		Cattle	Yes	0.71	1.53	1.00	2.34	1.944	0.052
			No	0.29	Ref				

From light trap collections, closed eaves were associated with significantly lower numbers of *An. arabiensis* (RR = 0.57, 95% CI: 0.33–0.96, p = 0.033) while significantly more *An. arabiensis* were collected in houses where some but not all residents of the household used a net the night before compared to houses where no one used a net (RR = 2.17, 95% CI: 1.02–4.62, p = 0.045). The number of *An. arabiensis* collected by PSC also was significantly lower in houses with closed eaves compared to those with open eaves (RR = 0.34, 95% CI: 0.18–0.67, p = 0.002). No other comparisons were statistically significant.

#### Sporozoite infection rates

**Figure 4:**
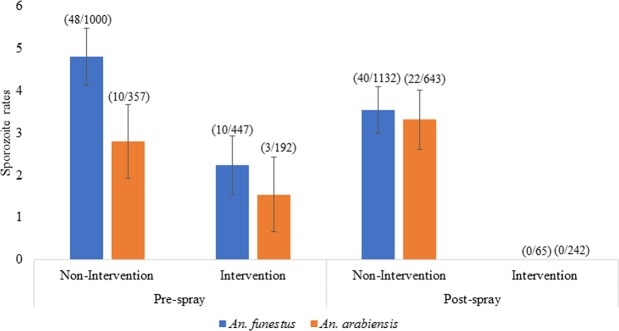
Sporozoite rates in *An. funestus* and *An. arabiensis* in sprayed and unsprayed areas, pre- and post-IRS.

Sporozoite infection rates in *Anopheles* mosquitoes were determined in intervention and non-intervention sites before and after IRS. Before IRS, 4.8% (48/1,000) of *An. funestus* were sporozoite positive in non-intervention sites compared to 2.2% (10/447) in the intervention sites whereas for *An. arabiensis*, sporozoite positivity rate was 2.8% (10/357) in non-intervention sites and 1.5% (3/192) in the intervention sites before IRS. Sporozoite infection rates for both species combined were not significantly different between intervention and non-intervention sites before IRS (RR = 0.27, 95% CI: 0.06–1.26, P = 0.09). After IRS, sporozoite infections were detected only in the non-intervention sites, where 3.5% (40/1,132) of *An. funestus* and 3.3% (22/643) of *An. arabiensis* were positive. (Fig. 4).

#### Vector biting behaviour

**Figure 5:**
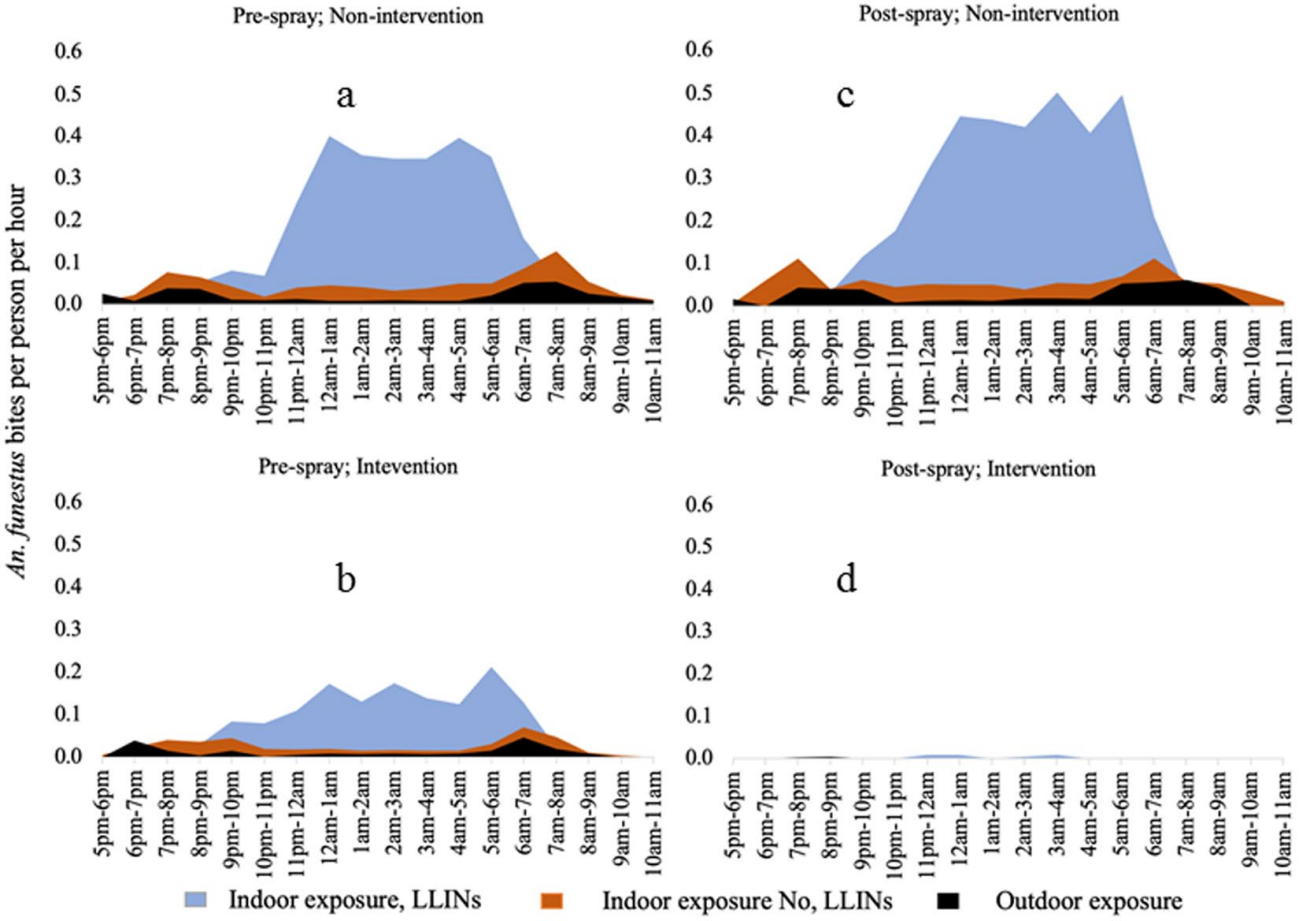
Profiles of biting by *An. funestus* experienced by the human population in intervention and non-intervention sites before and after IRS. The black area represents biting that occurs outdoors, the dark red represents biting that occurs indoors when people are away from their bed nets and the blue represents biting that occurs while people are asleep.

We estimated the exposure of humans to the risk of mosquito bites based on their observed behaviour and time and location of *An. funestus* biting. The numbers of *An. arabiensis* were insufficient to be included in the analysis. Over 70% of people within the study area were observed to be outdoors at 17:00, the beginning of mosquito collection. The number of people outdoors declined steadily over time with an increase in the number of individuals observed indoors, either in the living room (indoors not asleep) or in the bedroom (indoors and in bed). Over 90% of the people were observed to be indoors and in bed between 23:00 and 05:00 (Supplemental Fig. 4). In both intervention and non-intervention sites, before IRS, exposure to *An. funestus* was estimated to occur mostly, although not exclusively, indoors, late at night when people were asleep (Fig. 5a,b). In the post-IRS period, no change in the estimated exposure to bites by *An. funestus* was observed in the non-intervention sites (Fig. 5c). However, in the intervention sites, the risk of exposure to mosquito bites was nearly zero post-IRS (Fig. 5d). The relative proportion of bites by *An. funestus* increased both indoors and outdoors at dawn (05:00 am–08:00 am), corresponding to the time when most individuals woke up. Low levels of biting continued until 11:00 am when collection ceased.

#### Blood meal type

**Figure 6:**
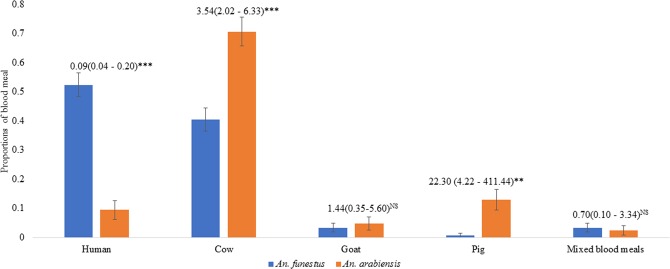
Comparison of mammalian host blood meal type between *An*. *funestus* and *An. arabiensis* (Numbers tested; *An. funestus*- 61 cow, 5 goats, 79 humans, 1 pig and 5 mixed and *An. arabiensis* – 60 cows, 4 goats, 8, humans, 11 pigs and 3 mixed).

Blood meal analysis using mosquitoes collected by PSC was conducted on 236 fed *Anopheles* mosquitoes, 151 *An. funestus* and 85 *An. arabiensis*. *An. funestus* fed mostly on humans 52.3% (79/151), followed by cattle 40.4% (61/151), goat 3.3% (5/151), pig 0.7% (1/151) and mixed-blood meals 3.3% (5/151, 2 human/cow, 2 human/goat and 1 human/pig). *An. arabiensis* had fed mostly on cattle blood 70.6% (60/85), followed by pig 12.9% (11/85), human 9.4% (8/85), goat 4.7% (4/85) and mixed-blood meal, human/goat 1.9% (1/85) (Fig. 6).

#### Malaria case count

A total of 137,972 laboratory test records from patients attending the out-patient departments were extracted from the five health facilities. For the two-year period before IRS (January 2015–February 2017), malaria test positive proportions were similar at 33.2% (18,036/54,404) in intervention and 33.3% (12,920/38,835) in non-intervention sites respectively. For the post-IRS period (March 2017–May 2018), the test positivity rates were 30.4% (6,347/20,882) in the non-intervention sites and 20.6% (4905/23,851) in the intervention sites.

**Figure 7:**
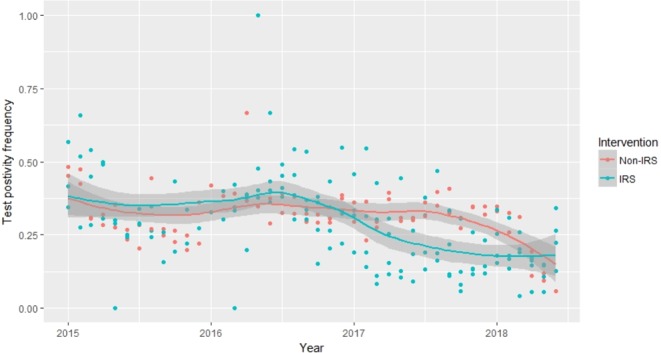
Health facility laboratory test positivity rates among febrile out-patients in Homa Bay (non-intervention) and Migori (Intervention) covering two years pre-IRS and over one-year post-IRS.

ARIMA analysis of malaria case counts for health facilities within IRS areas showed a reduction in malaria cases at the facilities post-IRS. Estimated mean monthly malaria cases in Rongo sub-county hospital dropped by 44% from 323 cases per month before IRS to 178 after IRS [mean difference = −142; 95% CI: −236 to −48; P = 0.003]. A similar reduction in mean monthly malaria cases with a 65.0% drop from 301 before IRS to 78 cases after IRS [mean difference = −196; 95% CI: −345 to −47; P = 0.01] was observed in Uriri sub-county hospital. In Nyatike sub-county hospital, the mean monthly malaria cases dropped by 47.4% from 118 cases before IRS to 72 after IRS [mean difference = −56; 95% CI: −123 to 11; P = 0.1]. For the two health facilities within non-IRS sites, no significant changes in malaria case counts were observed post-IRS, [Ndhiwa hospital: mean difference = −82; 95%CI: −230 to 65; P = 0.3; Marindi hospital: mean difference = 9.3; 95%CI: −132 to 151; = 0.9]. A plot of positive malaria cases over time, before and after IRS, showed a decline in the number of cases detected at facilities within sprayed areas compared to those in unsprayed regions (Fig. 7).

## Discussion

Our results demonstrate a significant reduction in *An. funestus* indoor resting densities, biting rates and sporozoite infections, as well as a decline in malaria test positivity rates and case-count at health facilities after one round of Actellic 300CS IRS in Migori County, western Kenya. Human biting rates and sporozoite infections in *Anopheles* mosquitoes are the most direct entomological measures of malaria infection risk. We observed moderate biting and sporozoite rates in both intervention and non-intervention sites before IRS and the unsprayed sites after IRS. However, after IRS, *An. funestus* biting rates were nearly zero, and no sporozoite infections were detected post-IRS. Susceptibility tests confirmed that the major vector species, *An. arabiensis* and *An. funestus*, were both resistant to pyrethroid insecticides but were susceptible to pirimiphos-methyl, the active ingredient in Actellic 300CS.

Similar reductions in *An. funestus* populations to near elimination were observed in the Asembo Bay area of western Kenya following the scale-up of pyrethroid-treated nets^5^ although *An. funestus* later returned as the primary malaria vector in the region presumably due to the development of pyrethroid resistance^34^. The complete elimination of *An. funestus* following effective IRS campaigns have been reported in South Africa, Mauritius and the Pare/Taveta area of Tanzania and Kenya^49,50^. *An. funestus* is particularly sensitive to effective insecticides used in IRS, probably due to the highly endophilic and anthropophilic nature of the species^51–53^, leading to high levels of exposure to treated surfaces.

In contrast, *An. arabiensis* indoor resting densities, human-biting rates, and sporozoite infection rates all reduced only marginally in sprayed areas post-IRS. With the decline in *An. funestus*, *An*. *arabiensis* became the predominant vector species in the sprayed areas (although the densities of *An. arabiensis* did not increase). IRS has a limited impact on the population of *An. arabiensis*, despite full susceptibility to pirimiphos-methyl in WHO susceptibility tests. This lesser impact of IRS on *An. arabiensis* is therefore unlikely to be due to insecticide resistance but may be attributable to the behaviour of this species. Blood-meal host analysis showed that *An. arabiensis* fed more frequently on cattle than humans, unlike *An. funestus* that fed more frequently on humans. This finding is supported by a previous study in western Kenya that reported *An. arabiensis* fed predominantly on cattle (65% of blood meals on cattle; 22% mixed bovine/human; 13% human)^8^. Furthermore, results from a deterministic model, developed using data from Kilombero, Tanzania, suggested that *An. arabiensis* fed outdoors on both humans and cattle and rapidly exited houses without fatal exposure to insecticidal nets or IRS^54^. A recent trap evaluation in western Kenya observed over sevenfold more *An. arabiensis* collected by cow odour compared to human odour outdoor^55^. Therefore, it is likely that a significant population of *An. arabiensis* rests predominantly outdoors and feeds primarily on cattle, but occasionally bites humans and transmits malaria, albeit less efficiently than the more anthropophilic vector *An. funestus*. Therefore, it is possible that the population of *An. arabiensis* collected indoors by light traps and PSC represents only a proportion of a larger outdoor population. These factors may explain the lesser impact of IRS on *An. arabiensis*.

IRS with Actellic 300CS had a prolonged residual activity of at least ten months post-IRS, as measured by wall bioassays. As spraying was conducted in February, the insecticide provided protection throughout the periods of highest malaria transmission during the long (April–June) and short (October–November) rainy seasons. This long residual efficacy of Actellic 300CS makes the insecticide particularly useful in providing all-year-round protection with just one spray round each year. Similar prolonged residual activity of Actellic 300CS and control of pyrethroid-resistant mosquitoes have been reported in other countries^32^. As this is the first report of the efficacy of IRS with a non-pyrethroid insecticide in Kenya in the wake of pyrethroid resistance, strict adherence to the insecticide resistance management strategy^27,28^ is needed. The Kenya National Malaria Control Program (NMCP) has a responsibility to fast track registration of additional non-pyrethroid classes of insecticides to enable rotation of insecticide in IRS for effective resistance management.

This study presents three novel findings that are crucial for malaria control and elimination in Kenya. Our study demonstrated that a single spray-round with Actellic 300CS is effective for year-round control of malaria in the region. Well-timed IRS, just before the peak malaria transmission, suppressed the local malaria vector populations through both the long and short rain periods, hence reducing disease transmission even during the peak transmission seasons that coincide with the long and short rain seasons. Second, the impact of IRS on *An. arabiensis* was less than that on other vectors, making it the dominant malaria vector sustaining residual transmission. While IRS was effective in controlling *An. funestus, An. arabiensis* would require alternative control approaches for malaria elimination. Finally, the study demonstrates late morning biting by *An. funestus*, a phenomenon not before investigated in the region. This presents a new challenge facing vector control given that the tools currently deployed for vector control are limited to the household and mostly only to the times when people are asleep (LLINs).

Biting by *An. funestus* in the intervention and non-intervention areas before IRS and non-intervention areas after IRS occurred mostly indoors late at night corresponding to the period when most people were indoors and in bed. Late night, indoor biting by *An. funestus* has been previously reported, dating back to the pre-bed net era. For instance, in 1975, 94% of *An. funestus* were observed to bite after midnight, with another a peak in the hours before dawn in Kano plain of western Kenya^56^. Similar late night, indoor biting was observed in the same study area in 1996^53^ and more recently the vector species have been reported to persistently bite indoors, late in the night despite high coverage in insecticide-treated nets^52^. While all these studies observed a biting peak at dawn, the collections ceased at 07:00 hours. With extended collections to monitor *An. funestus* biting in the morning, we observed biting until 11:00 hours. Similar findings of day-biting *An. funestus* in the presence of LLINs have been recently reported in Senegal^9^ and Benin^57^. This observed extended biting behaviour in *An. funestus* not previously investigated may potentially undermine the effectiveness of LLINs as people may be exposed to mosquito bites while away from the protection of their bed nets. However, one round of IRS with Actellic 300CS substantially reduced the number of *An. funestus* collected and it was not possible to detect biting either indoors or outdoors in the sprayed areas post-IRS.

In an analysis of risk factors associated with the indoor occurrence of mosquitoes, fewer *An. funestus* and *An. arabiensis* were collected by light traps in houses with closed eaves. Similar results were observed for *An. arabiensis* collected by PSC. Open eaves are known to be the main route for indoor entry of *Anopheles* mosquitoes^11,58^ and blocking them has been demonstrated to be effective in preventing *Anopheles* house entry^59^. Closing eave spaces or deploying vector control tools in these spaces may present an additional intervention to the current vector control tool kit for reducing the indoor occurrence of mosquitoes in addition to IRS and LLINs. However, new tools targeting outdoor *Anopheles* populations, in particular, *An. arabiensis*, are needed to control vector species that are less well controlled by interventions that target the indoor environment such as IRS and LLINs.

Reductions in malaria cases at the health facilities within sprayed areas post-IRS provided further evidence of the impact of a single round of IRS on malaria transmission. Health facility-based surveys of malaria cases in febrile patients have been useful as part of rapid analysis of changes in local malaria epidemiology^60–63^. Malaria infection is highly correlated with febrile cases reported at the health facilities^62^. Furthermore, a systematic review of febrile illness over 20 years in sub-Saharan Africa reported a dramatic reduction in the proportion of fevers associated with *Plasmodium falciparum* malaria^64^. Consequently, reductions in malaria cases likely contribute considerably to the reductions in febrile illnesses presenting at health facilities. The use of routine Health Management Information System (HMIS) data to evaluate malaria control interventions^63^, however, suffers from incompleteness in reporting and variation in the utilization of the health system^65^. We extracted data from the primary records, health facility laboratory registers and observed a reduction in confirmed malaria cases in health facilities within sprayed areas post-IRS, with no change in the number of cases detected at the facilities in control regions. Reduction in *An. funestus* densities, sporozoite rates and man-biting rates coupled with reduced malaria cases following one round IRS with pirimiphos-methyl provide compelling evidence of the effectiveness of IRS in malaria transmission reduction when implemented with an effective insecticide to which mosquito populations are susceptible.

This study compared entomological indices and malaria test positivity rates between one district receiving IRS in addition to LLINs and the neighboring districts receiving only LLINs. This approach was driven by the fact that it was an effectiveness study rather than a highly controlled study to assess efficacy. However, the study does raise questions about the optimal approach to evaluate vector control interventions such as IRS or larval source management which target population-wide suppression of malaria vectors and where the scale of the study may affect the magnitude with which the results can be generalized. While cluster randomized trials might be ideal for the evaluation of these tools, the flight range of mosquitoes should be accounted for as migration from outside clusters and/or spill-over effects from intervention clusters into control clusters may bias results towards the null^66^. However, larger clusters, in addition to being logistically unfeasible, may result in clusters that are spatially distant and less similar which may also bias the results. In this study, the non-random selection of areas for IRS, the low number of clusters and their spatial distance are a potential limitation of the study. However, the before and after design coupled with multiple endpoints indicating similar trends in mosquito densities and malaria-related outcomes strongly suggest that the results are robust.

## Conclusion

IRS with pirimiphos-methyl was highly effective for the control of indoor biting and indoor resting, pyrethroid-resistant *An. funestus* and resulted in substantially reduced numbers of this primary vector species coupled with reduced malaria cases. Due to the long residual effect of pirimiphos-methyl, it was possible to achieve year-round protection with a single round of IRS. Sustaining these gains is a priority for the Kenya NMCP and development partners and IRS should continue to be implemented to sustain the impact on *An. funestus*. However, there was less of an impact of spraying on *An. arabiensis* populations, likely due to their exophilic nature. Additional control measures are needed to control outdoor biting and resting *An. arabiensis*.

## Supplementary information

Supplementary Information.

## Data Availability

All data generated or analyzed during this study are included in this published article and its Supplementary Information files.
