# Supplementary material for: Impact of indoor residual spraying with pirimiphos-methyl (Actellic 300CS) on entomological indicators of transmission and malaria case burden in Migori County, western Kenya
Source: Sci Rep. 2020 Mar 11;10:4518. doi: 10.1038/s41598-020-61350-2 (PMC7066154; doi:10.1038/s41598-020-61350-2)
Supplement: Supplementary file 1 — Supplementary Information. [file 41598_2020_61350_MOESM1_ESM.pdf]

Impact of indoor residual spraying with pirimiphos-methyl (Actellic 300CS) on entomological indicators of transmission and malaria case burden in Migori County, western Kenya

Authors
Bernard Abong'o[1,2,3], John E. Gimnig[4], Stephen J. Torr[2], Bradley Longman[1], Diana Omoke[1], Margaret Muchoki[1], Feiko ter Kuile[2], Eric Ochomo[3], Stephen Munga[3], Aaron M. Samuels[4], Kiambo Njagi[5], James Maas[2], Robert T. Perry[6], Christen Fornadel[7], Martin J. Donnelly[2], Richard M. Oxborough[8]

Institutional affiliation
[1]Abt Associates Inc. PMI VectorLink Project, Whitehouse, Milimani, Kisumu, Ojijo Oteko Road, P.O. Box 895-40123, Kisumu, Kenya
[2]Liverpool School of Tropical Medicine, Pembroke Place, Liverpool, L3 5QA, UK
[3]Centre for Global Health Research, Kenya Medical Research Institute, P.O. Box 1578, Kisumu, Kenya
[4] Division of Parasitic Diseases and Malaria, Center for Global Health, Centers for Disease Control and Prevention, Atlanta, GA 30333, USA
[5] Kenya National Malaria Control Programme (NMCP), Ministry of Health, PO Box 19982, Kenyatta National Hospital, Nairobi 00202, Kenya
[6] The United States, Presidents Malaria Initiative (PMI), US Embassy Nairobi, United Nations Avenue, Nairobi, Kenya
[7]The United States, Presidents Malaria Initiative (PMI), US Agency for International Development, Washington, DC, USA
[8] PMI VectorLink Project, Abt Associates 6130 Executive Blv, Rockville, MD 20852, USA

Supplementary figures

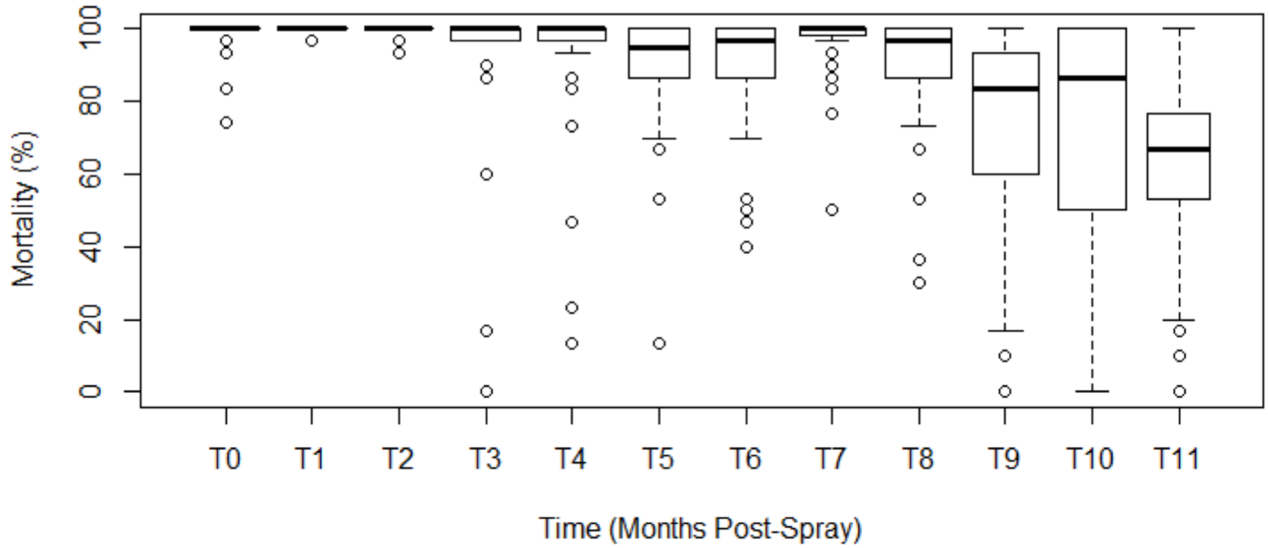

Supplemental Fig. 1. 24-hour mortality rates of susceptible *An. gambiae s.s*. exposed to mud and concrete sprayed walls over eleven months post-IRS. Distribution of mortality rates by box-whisker plots showing median values and interquartile ranges. Data were pooled for all sites.

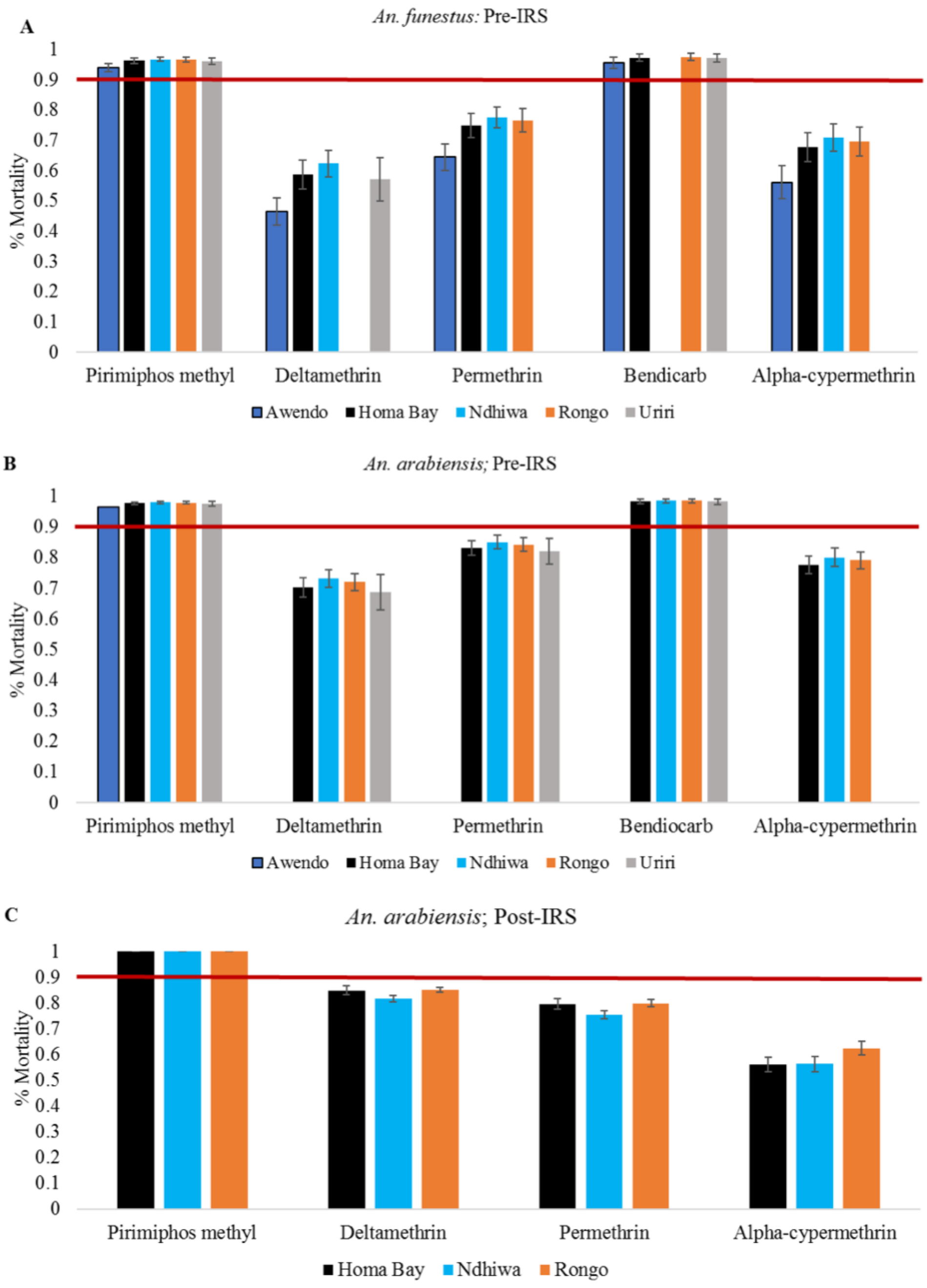

Supplemental Fig 2. 24-Hour mortality of *An. funestus* and *An. arabiensis* following exposure to pirimiphos-methyl, deltamethrin, permethrin, bendiocarb, and alpha-cypermethrin pre-IRS (Fig 2A and B) and post-IRS (Fig 2C) in WHO susceptibility test.

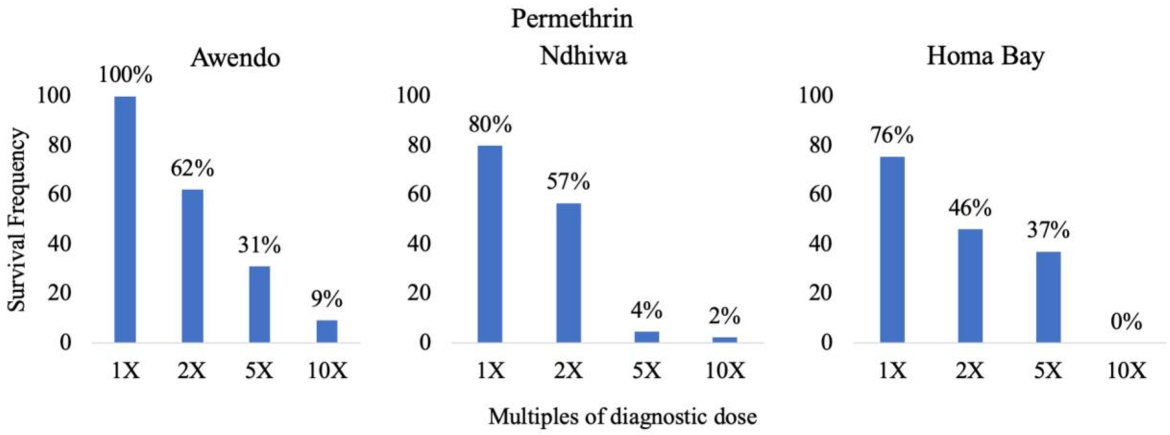

Supplemental Fig 3. 30 minutes survival frequency of *An. arabiensis* from Awendo, Ndhiwa, and Homa Bay sub-counties, exposed to multiples of diagnostic doses of permethrin.

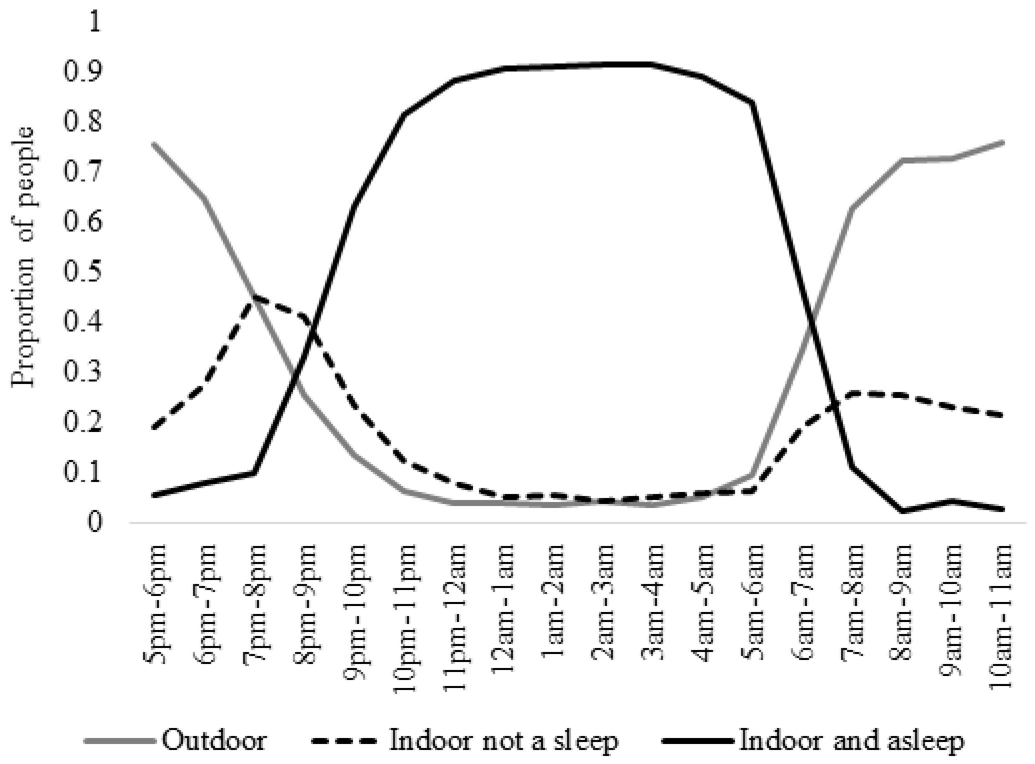

Supplemental Fig 4. Proportions of people within the study area at different locations during HLC collection.

Supplementary Tables

Supplemental Table 1. Model table for *Anopheles funestus* collected in light traps. Conditional estimates for the effect of pre- versus post-spray conditional on IRS status are also included.

**Parameter**	**Level**	**Risk Ratio**	**Lower CL**	**Upper CL**	**t-value**	**P-value**
Intercept	Interceptor	0.4	0.2	0.8	-2.841	0.013
Period	Post Spray	0.98	0.69	1.38	-0.127	0.899
	Pre Spray	Ref				
Status	Intervention	0.64	0.28	1.45	-1.063	0.288
	Control	Ref				
Period*Status	PostSpray*Intervention	0.12	0.07	0.21	-7.513	<0.001
	PostSpray*Control	Ref				
	PreSpray*Intervention	Ref				
	PreSpray*Control	Ref				
Net Use	All under net	1.11	0.77	1.6	0.576	0.565
	Some under net	1.2	0.77	1.86	0.816	0.415
	None under net	Ref				
Eaves	Closed	0.68	0.48	0.96	-2.174	0.030
	Partially open	0.84	0.56	1.27	-0.817	0.414
	Open	Ref				
Cattle	Yes	1.62	1.22	2.13	3.395	0.001
	No	Ref				
Period (Conditional on IRS)	Post Spray	0.12	0.07	0.19	-8.615	<0.001
	Pre Spray	Ref				
Period (Conditional on non-IRS)	Post Spray	0.98	0.69	1.38	-0.127	0.899
	Pre Spray	Ref				

Supplemental Table 2. Model table for *Anopheles funestus* collected by pyrethrum spray catches. Conditional estimates for the effect of pre- versus post-spray conditional on IRS status are also included.

**Parameter**	**Level**	**Risk Ratio**	**Lower CL**	**Upper CL**	**t-value**	**P-value**
Intercept	Interceptor	0.72	0.27	1.94	-0.704	0.493
Period	Post Spray	0.64	0.41	1.00	-1.945	0.052
	Pre Spray	Ref				
Status	Intervention	0.54	0.17	1.72	-1.047	0.296
	Control	Ref				
Period*Status	PostSpray*Intervention	0.06	0.03	0.13	-7.094	<0.001
	PostSpray*Control	Ref				
	PreSpray*Intervention	Ref				
	PreSpray*Control	Ref				
Net Use	All under net	0.96	0.61	1.5	-0.187	0.852
	Some under net	2.02	1.13	3.59	2.383	0.017
	None under net	Ref				
Eaves	Closed	0.8	0.5	1.3	-0.889	0.374
	Partially open	1.08	0.64	1.83	0.291	0.771
	Open	Ref				
Cattle	Yes	1.63	1.12	2.35	2.583	0.010
	No	Ref				
Period (Conditional on IRS)	Post Spray	0.04	0.02	0.07	-9.289	<0.001
	Pre Spray	Ref				
Period (Conditional on non-IRS)	Post Spray	0.64	0.41	1	-1.945	0.052
	Pre Spray	Ref				

Supplemental Table 3. Model table for *Anopheles arabiensis* collected in light traps. Conditional estimates for the effect of pre- versus post-spray conditional on IRS status are also included.

**Parameter**	**Level**	**Risk Ratio**	**Lower CL**	**Upper CL**	**t-value**	**P-value**
Intercept	Interceptor	0.03	0.01	0.07	-7.351	<0.001
Period	Post Spray	3.06	1.59	5.92	3.335	0.001
	Pre Spray	Ref				
Status	Intervention	1.79	0.55	5.76	0.972	0.331
	Control	Ref				
Period*Status	PostSpray*Intervention	0.45	0.2	1.01	-1.942	0.052
	PostSpray*Control	Ref				
	PreSpray*Intervention	Ref				
	PreSpray*Control	Ref				
Net Use	All under net	1.95	0.99	3.84	1.94	0.052
	Some under net	2.17	1.02	4.62	2.008	0.045
	None under net	Ref				
Eaves	Closed	0.57	0.33	0.96	-2.131	0.033
	Partially open	0.78	0.43	1.42	-0.814	0.416
	Open	Ref				
Cattle	Yes	1.33	0.89	1.98	1.383	0.167
	No	Ref				
Period (Conditional on IRS)	Post Spray	1.39	0.78	2.47	1.112	0.266
	Pre Spray	Ref				
Period (Conditional on non-IRS)	Post Spray	3.06	1.59	5.92	3.335	0.001
	Pre Spray	Ref				

Supplemental Table 4. Model table for *Anopheles arabiensis* collected by pyrethrum spray catches. Conditional estimates for the effect of pre- versus post-spray conditional on IRS status are also included.

**Parameter**	**Level**	**Risk Ratio**	**Lower CL**	**Upper CL**	**t-value**	**P-value**
Intercept	Interceptor	0.12	0.04	0.38	-3.954	0.001
Period	Post Spray	1.64	0.87	3.09	1.543	0.123
	Pre Spray	Ref				
Status	Intervention	1.16	0.3	4.44	0.214	0.831
	Control	Ref				
Period*Status	PostSpray*Intervention	0.36	0.16	0.82	-2.445	0.015
	PostSpray*Control	Ref				
	PreSpray*Intervention	Ref				
	PreSpray*Control	Ref				
Net Use	All under net	1.61	0.9	2.87	1.594	0.111
	Some under net	1.85	0.89	3.84	1.652	0.099
	None under net	Ref				
Eaves	Closed	0.34	0.18	0.67	-3.165	0.002
	Partially open	0.60	0.32	1.13	-1.583	0.114
	Open	Ref				
Cattle	Yes	1.53	1.00	2.34	1.944	0.052
	No	Ref				
Period (Conditional on IRS)	Post Spray	0.60	0.33	1.09	-1.681	0.093
	Pre Spray	Ref				
Period (Conditional on non-IRS)	Post Spray	1.64	0.87	3.09	1.543	0.123
	Pre Spray	Ref				
